# A novel prognostic signature of chemokines for survival and immune infiltration in kidney renal clear cell carcinoma

**DOI:** 10.7150/ijms.84940

**Published:** 2023-06-19

**Authors:** Jianming Weng, Zhiyong Huang, Qiang Li, Yuzhen Huang, Shunping Chen

**Affiliations:** Department of Pathology, ZhangZhou Affiliated Hospital of FuJian Medical University, Zhangzhou city, Fujian Province 363000, China

**Keywords:** kidney renal clear cell carcinoma, chemokine, tumour microenvironment, immune infiltration, immune checkpoint

## Abstract

**Objective**: Studies have revealed the alteration of chemokines in the tumour microenvironment in renal clear cell carcinoma (KIRC), which is closely related with immune infiltration and the prognosis of patients with KIRC. This research aims to comprehensively clarify the signature of chemokines in KIRC and the correlation between chemokines and immune infiltration in the TME of KIRC.

**Methods**: The chemokine expression in KIRC were investigated by using multiple multiomics and bioinformatics tools. Hub-chemokines that were significantly related with the cancer stage and survival were identified. The role of hub-chemokines in the tumor microenvironment of KIRC was further assessed by using enrichment analysis, cancer-related pathway and immune infiltration analysis.

**Results**: A total of 20 chemokines were significantly elevated in KIRC. Based on the correlation with KIRC stages and survival, 13 hub-chemokines were identified. Among the hub-chemokines, the high expression of CXCL2, CXCL5 and CXCL13 were related with worse survival of KIRC patients. The hub-chemokines were associated with the activation of multiple cancer-related signaling pathways. The functions of hub-chemokines were mainly enriched in chemokine-mediated signaling pathway, immunocytes chemotaxis and chemokine activity. CCL4, CCL5, CXCL9, CXCL10 and CXCL11 were related with various types immune infiltration such as CD8^+^T cell, neutrophil, B cell and dendritic cell. Using the hub-chemokine CXCL10, multiple immune checkpoints including LAG3, CTLA-4 and PD-1 were identified.

**Conclusion**: Our research sheds light on the chemokines and their important role in promoting the tumor microenvironment of KIRC. The findings could provide more data about the prognosis prediction and treatment targets for KIRC.

## 1. Introduction

Renal cancer represents one of the most frequently occurring primary tumors in the urological system, accounting for approximately 3% of adult malignant cancers 1. Among all the histological subtypes of renal cancer, kidney renal clear cell carcinoma (KIRC) is the most common subtype, constituting up to 80% of all cases 2. Despite that the majority of patients are at early stages of disease at diagnosis, up to one-third of them are diagnosed with metastases, and more than 25% might develop metastases after treatment 3. In spite of recent development in molecular targeted treatments including anti-vascular endothelial growth factors and mammalian target of rapamycin inhibitors, *etc.*, improving the prognosis of KIRC patients remains a critical clinical challenge 4. Therefore, investigating innovative potential biomarkers for prognostic estimation and individualized therapy is of utmost clinical importance.

The chemokines family are small secreted molecules that are important for inflammatory reactions and antitumour immunity. The chemokines can be produced by not only the macrophages, leukocytes and endothelial cells but also cancer cells. The chemokines can participate in the directional migration of immune cells such as T lymphocytes, monocytes and neutrophils, as well as modulate cancer-related angiogenesis and tumor cell metastasis 5, 6. The tumor microenvironment is composed of soluble and cellular components and various cell types including tumor cells and immune cells embedded in an altered extracellular. Research on cancers has revealed altered chemokines in the tumor microenvironment, thus influencing cancer cells proliferation and metastasis 7, 8. Yu *et al.* systematically examined the expression of CXC chemokines in colorectal cancer and observed that the expression of CXCL1-3, CXCL5, and CXCL8 were increased in colorectal cancers tissues than in colorectal tissues 9, suggesting that these chemokines can be used as potential treatment targets and prognostic indicators and that they might participate in the anti-tumor or tumorigenicity response. The cancer-promotion or -inhibition effects of chemokines are mainly due to the capacity to inhibit or enhance the reaction of the immune system 10. To date, multiple risk signatures have been investigated in KIRC to understand the prognostic value of genes related to inflammation. However, the role of chemokines in KIRC has not yet been fully verified.

Therefore, in the present study, we aimed to systematically analyze the expression of the chemokine family and its association with hallmarks of KIRC. We also verified the relationship between chemokines and immunocytes infiltration in the tumor microenvironment, which would be of help to further understand the chemokine family and improve therapeutic designs and the accuracy of prognosis for patients with KIRC.

## 2. Methods

**Figure 1:**
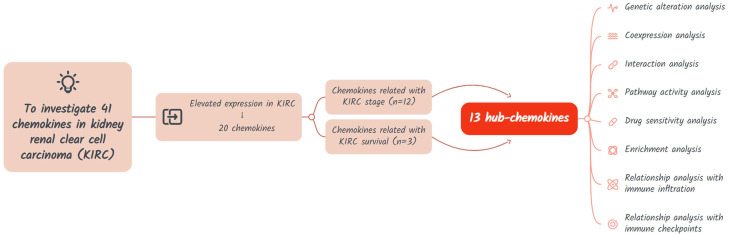
Flow chart of the study design.

The study design is shown in **Figure 1**. A total of 41 chemokines were investigated in this study. Firstly, we evaluated the expression of these chemokines in KIRC and identified those with elevated expression levels. Then the hub-chemokines were identified by analyzing the correlation of chemokines and KIRC stage and survival. Lastly, to clarify the role of these hug-chemokines, we further performed the genetic alteration analysis, co-expression analysis, interaction analysis, pathway activity analysis, drug sensitivity analysis, enrichment analysis, as well as the relationship analysis with immune infiltration and immune checkpoints.

### 2.1 Expression of chemokines in KIRC

We explored the mRNA expression of chemokines in KIRC in The Cancer Genome Atlas (TCGA) database by using GSCALite, a web server for gene set cancer analysis 11. The fold change and P values of chemokines in KIRC as compared with normal control were calculated and collected. Then, the chemokine expression levels were further investigated in pan-cancer by using Gene Expression Profiling Interactive Analysis, a web server for interactive analysis of 9,736 tumors and 8,587 normal samples from the TCGA and the GTEx projects 12. Furthermore, the corresponding expression levels of chemokines and their expression levels in four major pathological stages were analysed. Furthermore, the protein expression of chemokines were evaluated by the immunohistochemical (IHC) staining of KIRC tumor tissues in the Human Protein Atlas (HPA) database.

### 2.2 Investigation of chemokines in the prognosis of KIRC

We explored the prognostic significance of chemokines in KIRC by using the Kaplan-Meier method in GEPIA. The difference of survival curves were analysed by log-rank test. Both overall survival (OS) and disease-free survival (DFS) were analysed. The high and low expression group of each chemokine was separated by using the median expression value as the cut-of value.

To develop a prognostic signature based on chemokines, we downloaded the gene expression data of chemokines and survival information of patients with KIRC in The Cancer Genome Atlas (TCGA) database by the Genomic Data Commons (GDC) data portal. Univariate COX regression analysis was performed in the TCGA-KIRC cohort to explore the chemokines that were significantly (P < 0.05) related with KIRC survival by using the *rms* package in R software (version 4.3.0). LASSO penalty analysis was then performed to shrink the overfitting by using *glmnet* package in R. To construct a KIRC chemokine signature, multivariate COX regression model was constructed by using a stepwise process by using the *rms* packages in R. The hazard ratio (HR) for each chemokine was shown in a forest plot. The chemokine risk score for each individual was calculated based on the following formula: risk score =coefficient×(chemokine1 level) + coefficient×(chemokine2 level)+...+ coefficient×(chemokinen level). To assess the accuracy of the signature, all patients were split into the high and low risk groups using the median value of the chemokine risk score as the cut-off. The Kaplan-Meier curves of the two groups were plotted and compared by using the *survminer* package in R. The ROC (receiver operating characteristic) curves and calibration curves at 1-, 3- and 5-year were plotted to estimate the reliability of the chemokine risk score by using *pROC*, *timeROC* and *rms* packages in R.

### 2.3 The genetic alteration, co-expression and interaction analysis of chemokines in KIRC

The genetic alterations of chemokines in KIRC including amplication, deep deletion, missense mutation, truncating mutation, *etc.*, were investigated by using cBioPortal, an online tool for interactive exploration of multidimensional cancer genomics data sets. The information of KIRC samples was obtained from TCGA, Firehouse Legacy and Tokyo, Nat Genet 2013 dataset. The co-expression of chemokines in KIRC were explored in TCGA cohort. By using the protein-protein interaction (PPI) networks analysis online tools including search tool for the retrieval of interacting genes/proteins (STRING) and GeneMANIA, the potential interactions of the hub-chemokines were explored.

### 2.4 Cancer‑related pathway and drug sensitivity analysis

The cancer-related pathways and chemokines-related drug sensitivity in KIRC samples were investigated by using GSCALite. Several famous cancer related pathways were investigated including apoptosis, PI3K/AKT, TSC/mTOR, RTK, EMT, hormone ER, DNA damage response, hormone AR, RAS/MAPK and cell cycle pathways. When analyzing cancer-related pathway, the high and low expression levels of genes were divided by using the median expression level as the cut-off. The pathway activity scores were defined with t test and FDR value. As for the drug sensitivity analysis, the molecules were collected from the Genomics of Drug Sensitivity in Cancer (GDSC) database and analysed their relationship with hub-chemokines. The spearman correlation coefficients and log10(FDR) were calculated.

### 2.5 Enrichment analysis

For each hub-chemokine, the top five correlated genes were selected by using cBioPortal. The hub-chemokines and their correlated genes were integrated and used to perform enrichment analysis. Enrichment of hub-chemokines in KIRC was explored and visualized by using Database for Annotation, Visualization, and Integrated Discovery (DAVID), GeneMANIA and Metascape. In DAVID, the gene ontology (GO) including molecular function (MF), cellular component (CC) and biological process (BP) and Kyoto Encyclopaedia of Genes and Genomes (KEGG) pathway were analysed and visualized by using R. The hub-chemokines and their correlated genes were submitted to Metascape to perform pathway and process enrichment.

### 2.6 Immune infiltration analysis

The Tumor IMmune Estimation Resource (TIMER) was used for systematical analysis of immune infiltration related with chemokines 13. To further evaluate the prognostic role of immune infiltration in KIRC, we explored the clinical relevance of tumor immune subsets in a multivariable Cox proportional hazard model. The hazard ratios (HR) and statistical significance (p value) were calculated and collected. The Kaplan-Meier plots for immune infiltrates were drawn to visualize the survival differences.

### 2.7 The correlation of hub-chemokines with immune checkpoints

In consideration of the expression and prognostic significance of chemokines in KIRC, we selected specific hub-chemokines for the correlation analysis. The correlations between hub-chemokines and 12 common immune checkpoints were analysed in the TCGA database by using TIMER tool.

## 3. Results

### 3.1 Expression of the chemokines in KIRC

**Figure 2:**
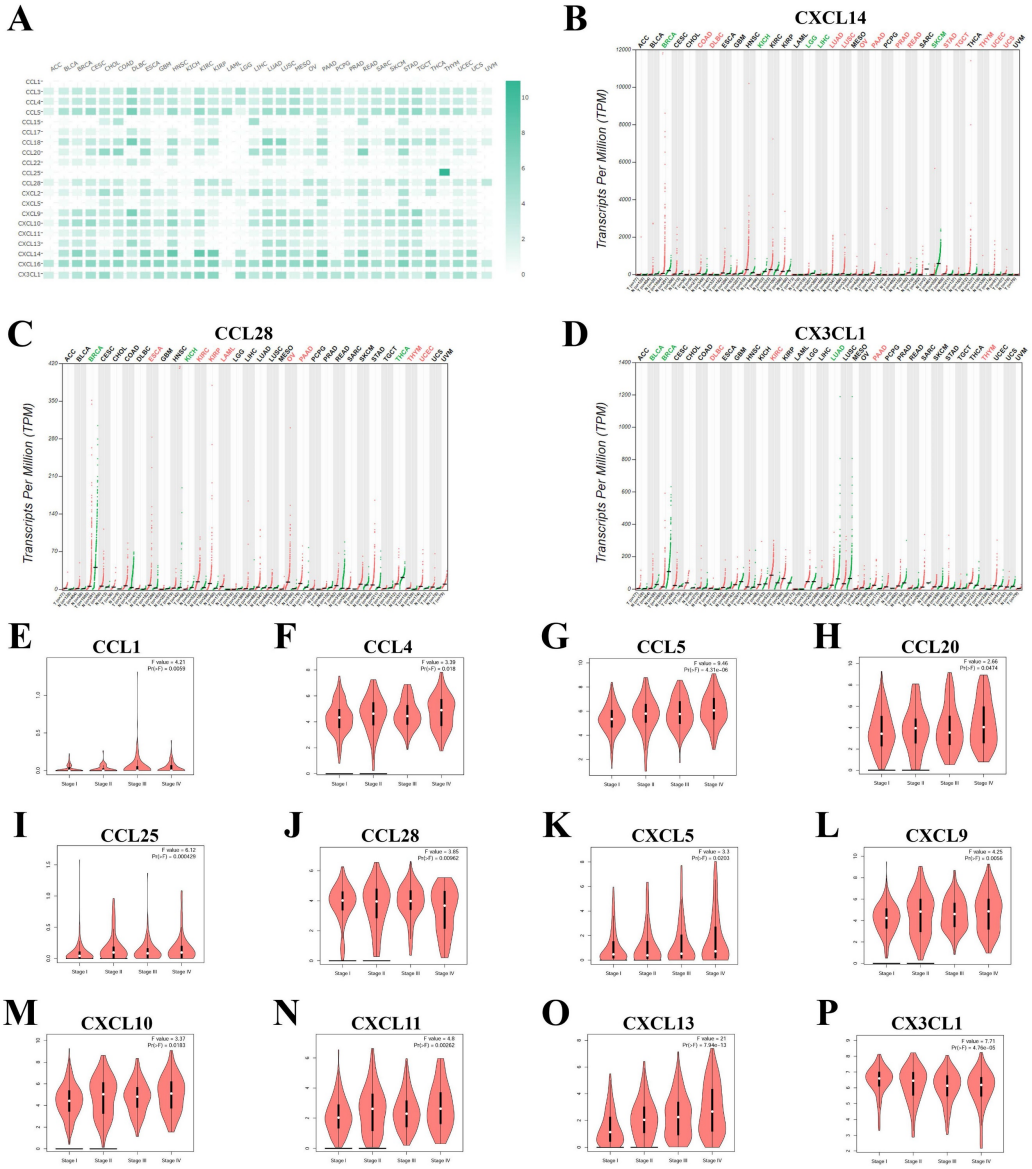
** The mRNA levels of chemokines in KIRC**. (A) The expression of elevated chemokines in KIRC were explored in multiple cancer types. The expression profile of (B) CXCL14, (C) CCL28 and (D) CX3CL1 were plotted. (E) to (P) The correlation of chemokines with different pathological stages of KIRC.

As shown in **Figure 1** and **Table 1**, a total of 41 chemokines were investigated in KIRC, and 20 of these chemokines showed significantly increased mRNA levels with fold change > 1 and P value < 0.05 as compared with normal tissue. CCL18 showed the highest fold change (90.77) and CXCL14 the lowest (1.6) (**Table 1**). The expression levels of these 20 increased chemokines in KIRC were also explored in pan-cancers (**Figure 2A**). Although the fold change of CXCL14 compared with normal tissue was the lowest among elevated chemokines, the absolute mRNA expression of CXCL14 was the highest in KIRC (**Figure 2A**). On the other hand, the CCL28, CXCL14 and CX3CL1 expression levels were higher than those in other tumours (**Figure 2A**). The expression profile plots of CCL28, CXCL14 and CX3CL1 expression in various tumours were shown in **Figure 2B, 2C** and **2D**.

The 20 elevated chemokines were also explored in different pathologic stages, and the following chemokines showed significant relevance with pathologic stages: CCL1, CCL4, CCL5, CCL20, CCL25, CCL28, CXCL5, CXCL9, CXCL10, CXCL11, CXCL13 and CX3CL1 (**Figure 2E** to **2P**). CCL5, CXCL13 and CX3CL1 showed the most significant correlation with pathologic stages. While the other 8 chemokines showed no significant relation with four major pathologic stages of KIRC (**Supplementary Figure S1**). Furthermore, we evalued the protein level of several chemokines in KIRC tumor tissues by using the IHC staining results in the HPA database. As shown in **Figure S2**, there were obvious elevation of IHC staining density in CCL4, CCL5, CXCL5 and CXCL13. The expression of CX3CL1 and CXCL11 showed high level of positive staining in normal tissues, and the changes in KIRC were not significant.

### 3.2 Chemokines correlated with the prognosis of KIRC

**Figure 3:**
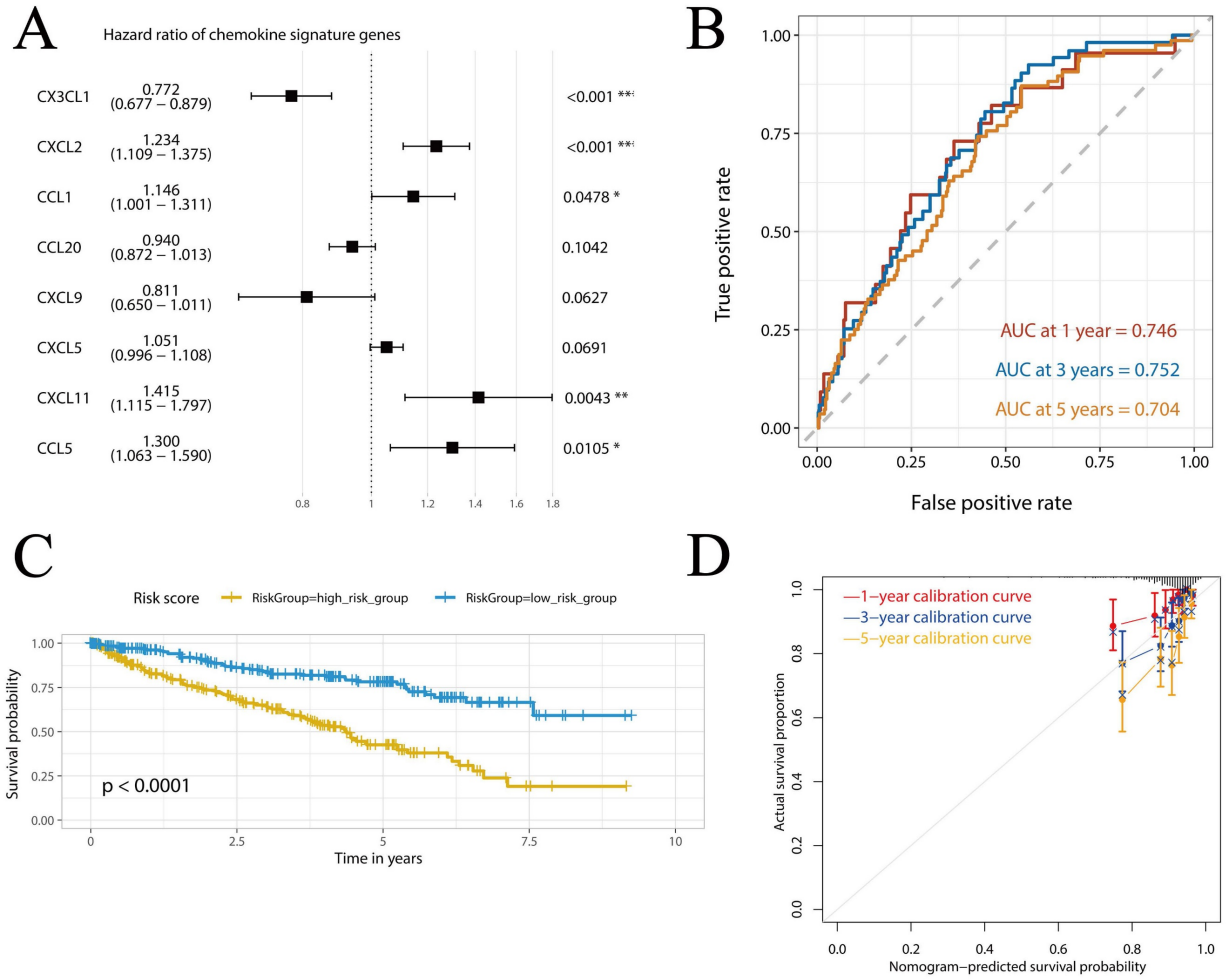
** Construction and verification of the chemokine risk score model in TCGA-KIRC cohort.** (A) Forest plot showing the hazard ratios of the chemokines. (B) ROC curves of the chemokine risk score at 1-, 3- and 5-year. (C) Survival curves of high- and low-chemokine risk groups plotted by K-M method. (D) Calibration curves of the chemokine risk score model at 1-, 3- and 5-year.

We assessed the association of 20 elevated chemokines with the OS and DFS of KIRC. Based on the data from GEPIA, we found that KIRC patients with higher expression of CXCL2 (HR = 1.6, P = 0.005, **Figure S3A**), CXCL5 (HR = 1.6, P = 0.002, **Figure S3B**) and CXCL13 (HR = 1.4, P = 0.026, **Figure S3C**) exhibited lower OS rate than those with lower gene expression. As for DFS, CXCL2 showed no significant relevance (P = 0.52, **Figure S3D**), while higher expression of CXCL5 (HR = 2, P < 0.001, **Figure S3E**) and CXCL13 (HR = 1.7, P = 0.007, **Figure S3F**) was also significant risk factor. As shown in **Figure 3A**, we further constructed a prognostic signature (risk score) based on the expression of several critical chemokines in KIRC including CX3CL1, CXCL2, CCL1, CCL20, CXCL9, CXCL5 and CXCL11. The risk score can be calculated as follows: socre = (-0.2593)*exp(CX3CL1) + 0.2106*exp(CXCL2) + 0.1359*exp(CCL1) - 0.0619*exp(CCL20) - 0.2098*exp(CXCL9) + 0.0495*exp(CXCL5) + 0.3475*exp(CXCL11) + 0.2628*exp(CCL5). As shown in **Figure 3B**, the area under curve (AUC) values at 1-, 3- and 5-year were all > 0.7, with AUC values at 1- and 3-year were 0.746 and 0.752, indicating that the chemokine risk score had a relatively good accuracy in predicting the survival of KIRC. Then, the TCGA-KIRC cohort was divided into high- and low-risk groups based on the cohort's median chemokine risk score value. The Kaplan-Meier curves of the two groups showed obvious dispersion with significantly higher survival rate in the low-chemokine-risk group (p < 0.0001, **Figure 3C**). Furthermore, the calibration curves showed good fitting of predicted survival and actual survival at 1-, 3- and 5-year (**Figure 3D**). The other chemokines showed no significant association with the prognosis of KIRC (P > 0.05 for all, **Supplementary Figure S4**).

### 3.3 The genetic alteration, co-expression and interaction analysis of hub-chemokines in KIRC

**Figure 4:**
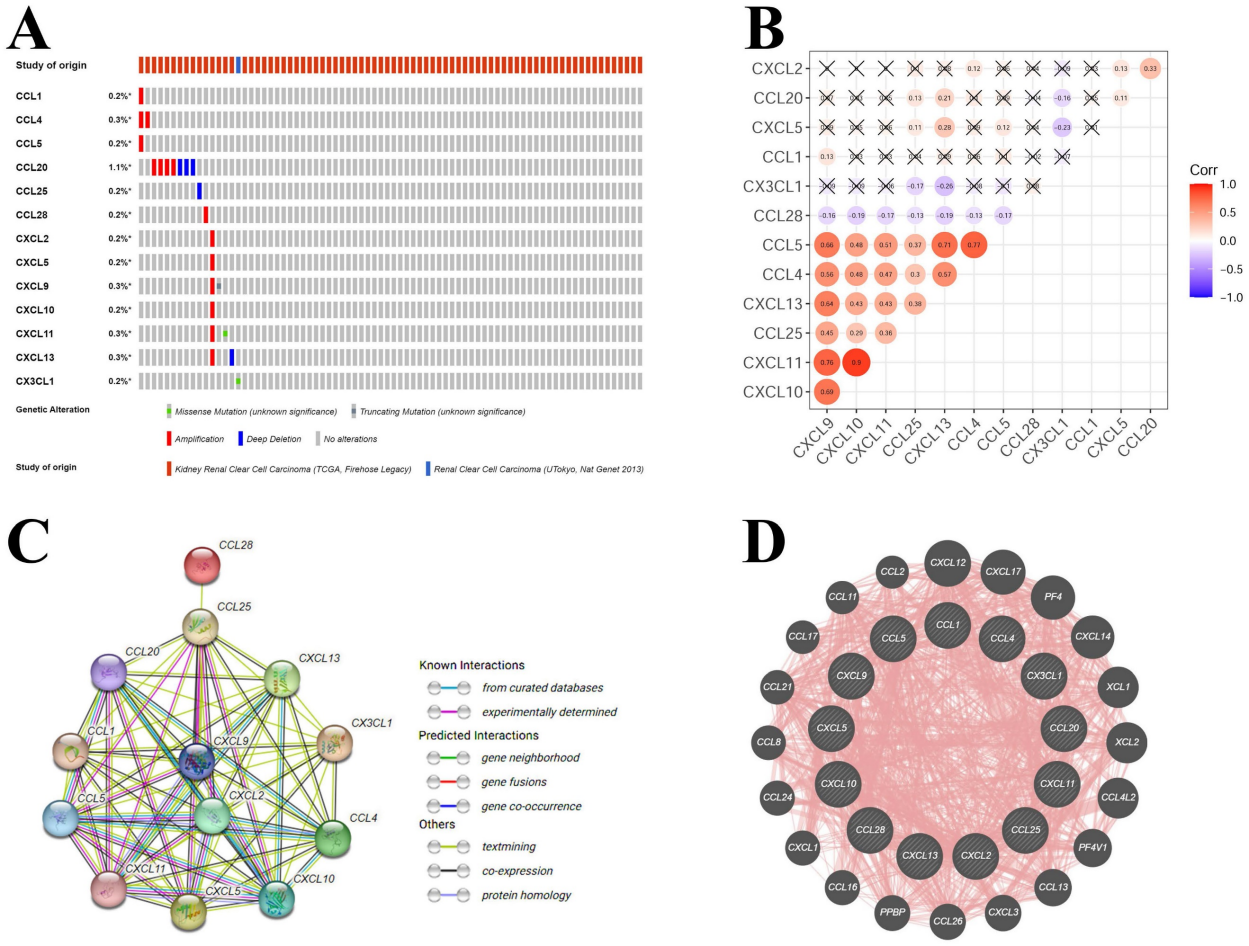
** Mutations and interactions of hub-chemokines in KIRC.** (A) Mutation information of hub-chemokines from cBioPortal. (B) Correlation between each hub-chemokine in KIRC. (C) PPI network from STRING. (D) Circular diagram of hub-genes from GeneMANIA.

Based on the results of correlation with KIRC pathologic stages and KIRC survival, a total of 13 hub-chemokines were identified: CCL1, CCL4, CCL5, CCL20, CCL25, CCL28, CXCL2, CXCL5, CXCL9, CXCL10, CXCL11, CXCL13 and CX3CL1. A comprehensive analysis of the molecular pattern of these hub-chemokines were performed. By using cBioPortal to analyze the genetic alterations, we found that the most frequent chemokine alteration in KIRC was gene amplification (**Figure 4A**). For chemokine CCL20, gene amplification was the most common alteration followed by deep deletion (**Figure 4A**).

The co-expression of hub-chemokines were shown in** Figure 4B**. CCL1 was not analysed due to limited number of valid expression values in the samples. Most of the hub-chemokines were significantly correlated. There was a very strong correlation of expression levels between CXCL10 and CXCL11, a high correlation among CCL4, CCL5, CXCL9 and CXCL11 (**Figure 4B**).

The PPI network analysis was conducted by using STRING (**Figure 4C**) and GeneMANIA (**Figure 4D**) to investigate the potential interactions among these hub-chemokines. The results of STRING analysis suggested that the functional enrichments in the hub-chemokines network were mainly related to leukocyte tethering or rolling, natural killer cell chemotaxis, cell-cell adhesion mediated by integrin, chemotaxis of lymphocyte and T cells, and chemokine receptor binding of CXCR3, CCR10, CXCR and CCR1, with 13 nodes and 64 edges (**Figure 4C**). The GeneMANIA analysis also indicated that the functions of hub-chemokines were predominantly associated with cellular response to chemokines, cytokine activity and chemokine receptor binding (**Figure 4D**).

### 3.4 Cancer‑related pathway and drug sensitivity analysis of hub-chemokines in KIRC

**Figure 5:**
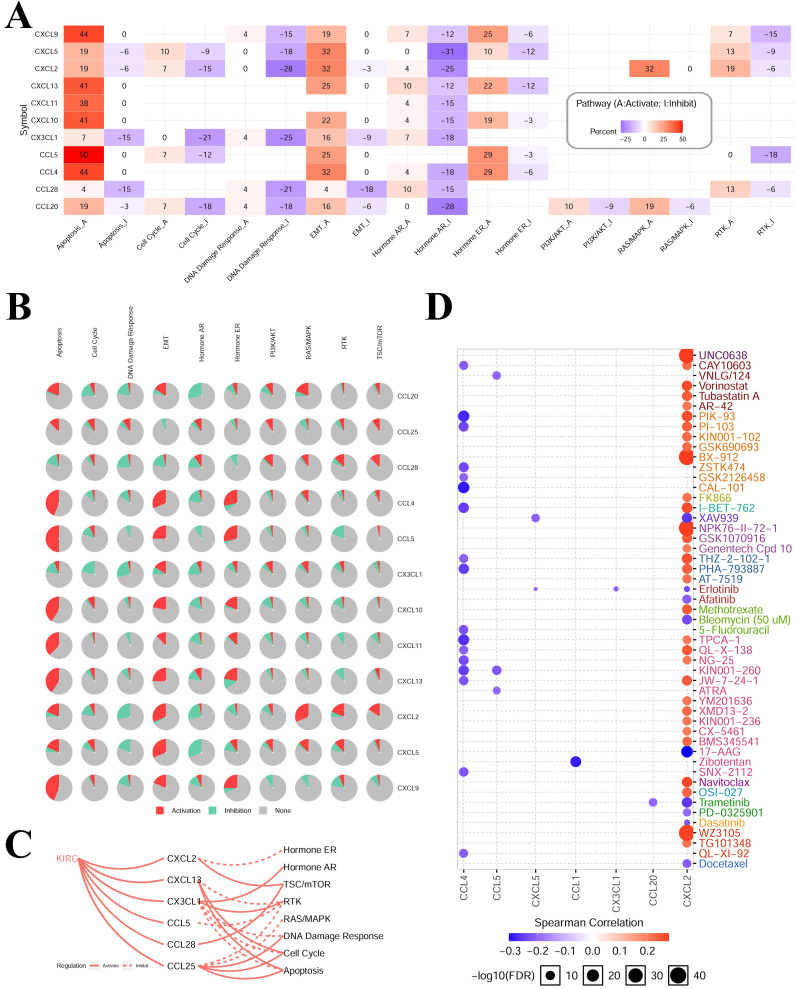
** Cancer-related pathways and drug sensitivity analysis.** (A) to (C) The role of chemokines in the activation and inhibition of most famous cancer-related pathways. (D) Drug sensitivity analysis of hub-chemokines in KIRC.

The results of cancer‑related pathway analysis indicated that most hub-chemokines were related with the activation of apoptosis, EMT, hormone ER pathways and the inhibition of hormone AR, DNA damage response pathways (**Figure 5A, 5B** and** 5C**). The result of drug sensitivity analysis indicated that the expression of CXCL12 was positively correlated with drug resistance (**Figure 5D**).

### 3.5 Enrichment analysis of chemokines in KIRC

**Figure 6:**
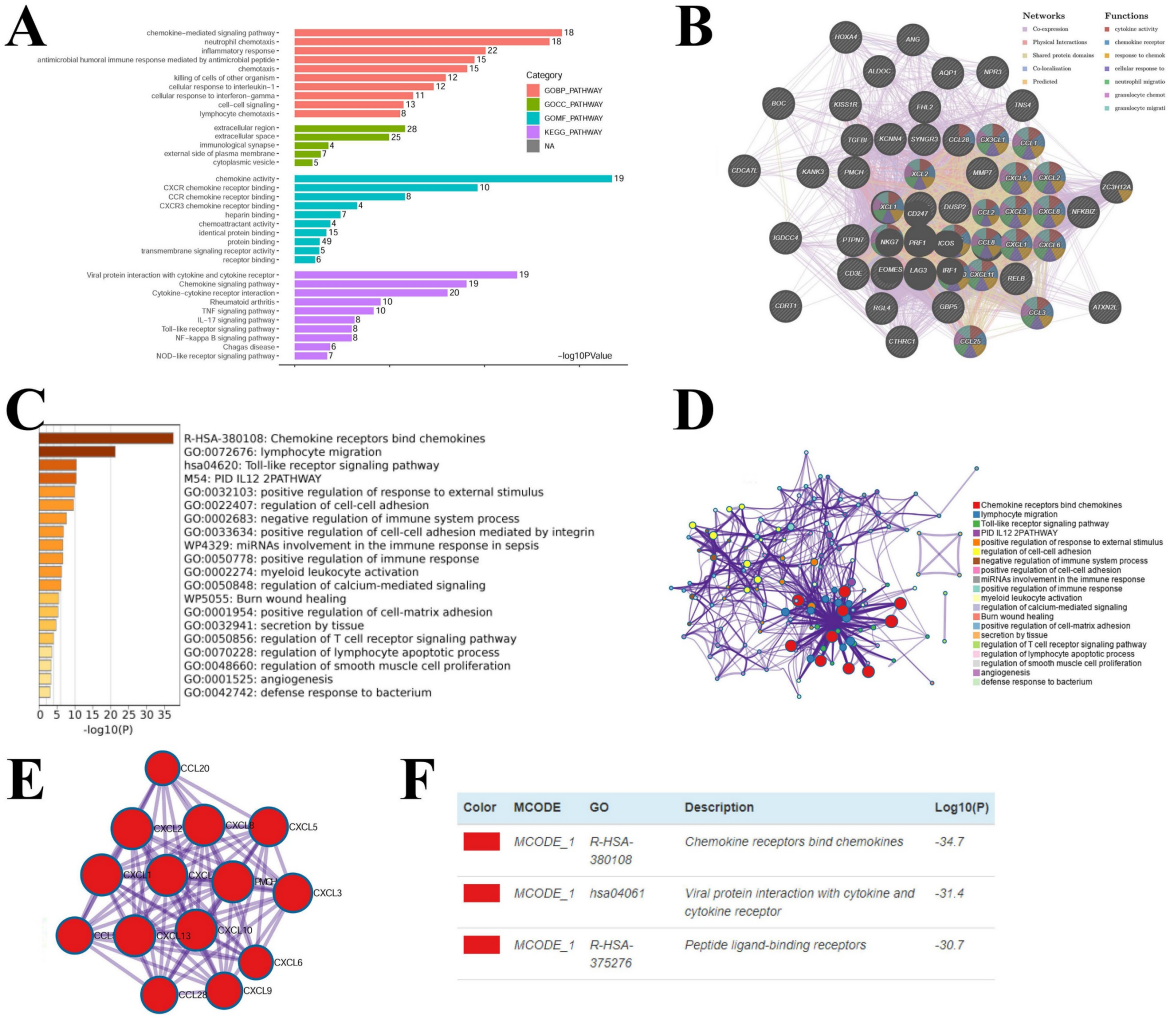
** Enrichment analysis of hub-chemokines and neighboring genes in KIRC.** (A) Bar plots of GO and KEGG enrichment. (B) The PPI network from GeneMANIA. (C) Bar graph of the top 20 enriched terms coloured by P values. (D) Network of enrichment terms coloured by cluster name. (E) PPI network of the hub-chemokines and (F) MCODE components.

Considering the critical function of chemokines in KIRC, we conducted enrichment analysis of the hub-chemokines. The top five correlated genes for each hub-chemokine were listed in **Table 2**. GO functional enrichment analysis suggested that the hub-chemokines were principally associated with chemokine-mediated signaling pathway, neutrophil chemotaxis and chemokine activity (**Figure 6A**), while in KEGG analysis we observed that the hub-chemokines were mainly enriched in viral protein interaction with cytokine and cytokine receptor (**Figure 6A**). PPI network analysis suggested that hub-chemokine function was predominantly involved in cytokine activity, chemokine receptor binding and response to chemokines (**Figure 6B**). The function of hub-chemokines and correlated genes were also validated by using Metascape. Similarly, the analysis by Metascape showed that the function was mainly enriched in chemokine receptors bind chemokines, lymphocyte migration and toll-like receptor signaling pathway (**Figure 6C** and** 6D**). In addition, the result obtained from MCODE showed that hub-chemokines played critical roles in chemokine receptors bind chemokines, viral protein interaction with cytokine and cytokine receptor and peptide ligand-binding receptors (**Figure 6E** and **6D**).

### 3.6 Correlation between immune infiltration and hub-chemokines in KIRC

**Figure 7:**
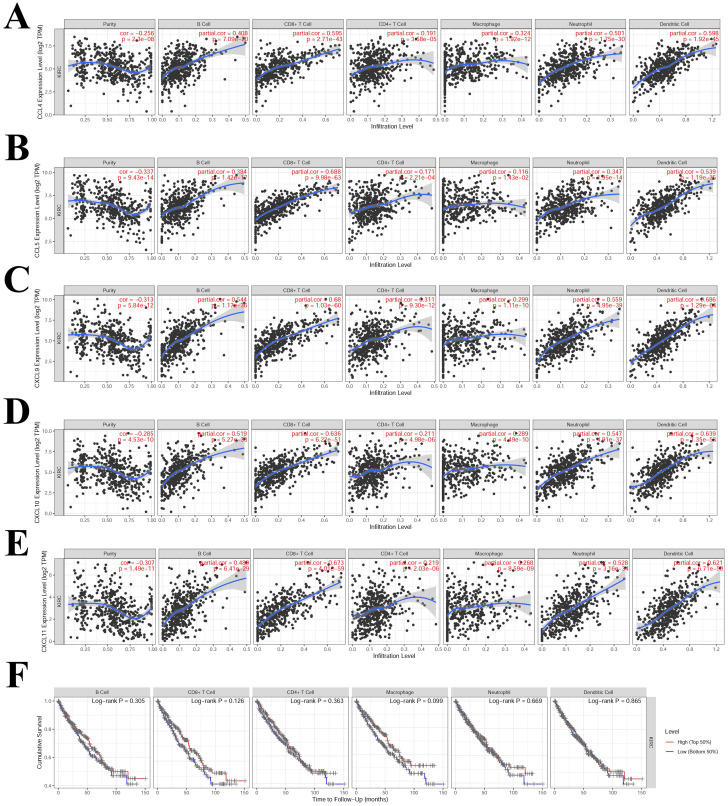
** Correlations analysis between hub-chemokines and different types of immune infiltration.** Scatter plots of (A) CCL4, (B) CCL5, (C) CXCL9, (D) CXCL10 and (E) CXCL11. (F) K-M curves of different types of immune infiltration in the survival of KIRC.

The TIMER results suggested that CCL4, CCL5, CXCL9, CXCL10 and CXCL11 had high correlation (correlation coefficient > 0.5, P < 0.05) with multiple immune cells infiltration (**Figure 7**). CCL4 was correlated with CD8^+^T cell, neutrophil and dendritic cell (**Figure 7A**). CCL5 was correlated with CD8^+^T cell and dendritic cell (**Figure 7B**). CXCL9, CXCL10 and CXCL11 were correlated with B cell, CD8^+^T cell, neutrophil and dendritic cell (**Figure 7C, 7D** and** 7E**). The correlations that were not significant between immune infiltration and hub-chemokines were shown in **Supplementary Figure S5**. The impact of each immune cell infiltration on survival was evaluated by using K-M method and the macrophage was the most close to statistical significance (P = 0.099). In the COX regression analysis of immune cells for survival of kidney renal clear cell carcinoma, the immune cells were included in the COX regression model and CD8^+^ T cell and macrophage showed significant impact on survival (P < 0.05).

### 3.7 Correlation analysis of immune checkpoints in KIRC

**Figure 8:**
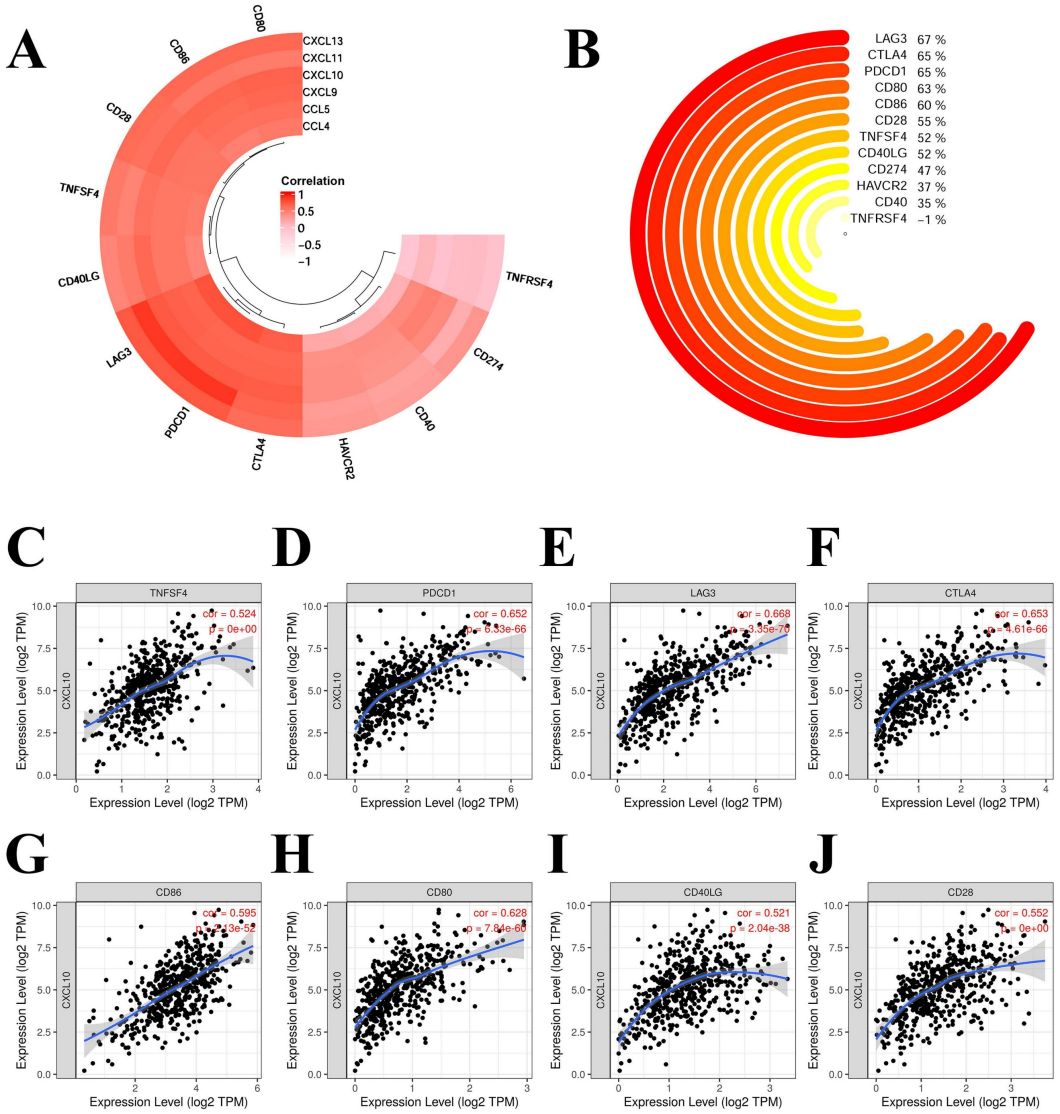
** Correlation of hub-chemokines and immune checkpoints in KIRC.** (A) Annular heatmap shows the correlation coefficients between selected hub-chemokines and immune checkpoints. (B) Circular plot shows the correlation of CXCL10 with these immune checkpoints. (C) to (J) Scatter plots show the expression level of CXCL10 and immune checkpoints.

We selected the following chemokines that were more correlated with the prognosis and immune infiltration of KIRC: CCL4, CCL5, CXCL9, CXCL10, CXCL11 and CXCL13. The correlation of chemokines and immune checkpoints in KIRC were explored. The immune checkpoints and chemokines with correlation coefficients greater than 0.75 were PDCD1 and CCL5 (0.884), LAG3 and CCL5 (0.874), PDCD1 and CXCL13 (0.761) (**Figure 8A**). CD80, CD86, CD28, LAG3 and PDCD1 showed high correlation with most of these chemokines (**Figure 8A**). We also analysed the correlation of CXCL10 with these immune checkpoints (**Figure 8B** to** 8J**). Most of these immune checkpoints were significantly correlated with CXCL10 with correlation coefficients > 0.5 and P < 0.001 (**Figure 8B** to** 8J**).

## 4. Discussion

In KIRC, the tumor microenvironment is capable of modulating carcinogenesis and tumor development and is tightly related with anti-cancer drug resistance, immune escape and survival 14-16. Multiple researches have found that chemokines are increased in various types of cancers and proven valuable in predicting prognosis 17. The chemokines play a critical part in the interactivity between the tumor microenvironment and tumor behaviors 18, 19. Therefore, verification of chemokines expression in KIRC and their relationship with tumor characteristics is of vital importance. In the present research, we clarified the significance of chemokines in KIRC by using multiomics and multiple bioinformatics tools. Generally, many chemokines were increased in KIRC and 13 hub-chemokines that were correlated with patient prognosis and tumor stage were identified. We also found that chemokines were involved in the activation of cancer-related signaling pathways, various types of immune infiltration and drug resistance. Additionally, the hub-chemokine CXCL10 was selected and several immune checkpoints of CXCL10 were identified.

Recently, CXCL10 has been identified as a prognostic biomarker and potential treatment target for KIRC. Qu *et al*. found that the expression level of CXCL10 in tumor tissues was significantly associated with the prognosis of patients with KIRC 20. Esteban *et al*. also observed that a high basal serum level of CXCL10 was significantly associated with reduced PFS and OS in a prospective cohort patient with metastatic renal cell carcinoma 21. C-X-C motif chemokine ligand 10 (CXCL10) is a 10-kDa secreted protein as a member of the CXC family, also known as interferon gamma-inducible protein 10 (IP-10). CXCL10 play import part in mediating the processes including leukocyte migration, adaptive immune, inflammatory response, hematopoietic and angiogenic reactions 22. CXCL10 is a chemokine that is frequently elevated during EGFR-TKI treatment in the tumor microenvironment of lung cancer. CXCR3 is a specific receptor of CXCL10, and most of the above CXCL10-mediated processes are through the CXCL10/CXCR3 signaling pathway. In the development of cancer, the autocrine CXCL10/CXCR3 pathway in cancer cells can induce the proliferation of cancer and metastasis by modulating cell adhesion, invasion, and migration activity 23, 24. The CXCL10/CXCR3 pathway has been found to show impact on the resistance to EGFR-tyrosine kinase inhibitors which was demonstrated by cytokine array analysis during *in vitro* coculture with tumor cells and activated PBMCs treated with EGFR-TKI 25. The importance of CXCL10/CXCR3 signaling pathway has also been tested in other types of tumors such as melanoma and colorectal carcinoma 26, 27. CXCL10 might also promote Th1 immune response and lead to increased number of CD8+ T cells in the inflamed tumor microenvironment 28. Therefore, the CXCL10/CXCR3 signaling pathway might be a potential treatment target and prognostic biomarker for KIRC.

The other hub-chemokines, especially those showed significant association with immune infiltration, were also important mediators in the process of cancer progression. For instance, CCL4 is found to act as an oncogene in KIRC since it is involved in shorter survival and more advanced tumor stage of KIRC 29. The high expression of CCL4 is also related with higher tumor mutation burden level in KIRC 29, which might further contribute to the remodeling of tumor microenvironment. On the other hand, another hub-chemokine, CCL5, is also found to be capable of inducing the immunosuppression in the tumor microenvironment and subsequent cancer progression and poor prognosis in KIRC *via* CCL5-dependent mast cell infiltration 30. The mechanism of CCL5 might also be associated with tumor-associated macrophages which can produce massive CCL5 in tumor tissues 31. The TME is associated with infiltration by multiple immune cells which is governed by chemokines 32. Tumor-associated macrophage (TAM) is the most abundant immune cell in the TME. Since many CD68+ TAMs are M2-polarised, and they also express CD163 in neuroendocrine-low tumor subsets, thus inducing an immunosuppressive microenvironment within the tumor nests 33. CCL5 can recruit the accumulation of macrophages and also regulate the epithelial-mesenchymal transition process *via* the PI3K/AKT pathway in KIRC cancer cells 31. Another hub-chemokine, CCL20, which showed the most gene alterations, was also found to be able to regulate the migration ability, epithelial-mesenchymal transition, and Akt phosphorylation in the human renal cell carcinoma cell line cells, and thus resulting in the poor prognosis of renal cancer 34. CCL20 can also be produced by tumor-associated macrophages in the tumor microenvironment of KIRC and then activate the cancer cells *via* Akt activation, followed by acquired migration activity and epithelial-mesenchymal transition 34. In brief, these hub-chemokines are critically important in regulating the tumor microenvironment of KIRC through various mechanisms and mediating the migration and proliferation of cancer cells.

The result of drug sensitivity analysis suggested that CXCL12 was positively correlated with drug resistance. It was recently found that CXCL12/CXCR4 is essential in the formation of the sphere in the renal organoid model, where endothelial and stem cell proliferation occurs collaboratively. CXCR4 and CXCL-12 can be assessed together in terms of the TME and used in microenvironment modeling in drug studies. The expression of CXCR4/CXCL12 was reported to be increased due to the hypoxic tumor environment 35.

Our research has several limitations. The hub-chemokines identified in this study and their association with tumor-related immune cells need to be further challenged with *in vitro* or *in vivo* experiments. Besides, all clinical cases of KIRC included in the current research were acquired from the TCGA database, therefore the potency of the chemokine risk score also needs to be validated by external databases.

## Conclusion

In summary, the present research reveals novel insights into the chemokines and their role in the tumor microenvironment of KIRC. The findings could present new clues and directions of the prognosis prediction and treatment targets for KIRC.

## Supplementary Material

Supplementary figures.

## Figures and Tables

**Table 1:** The mRNA levels of chemokines in kidney renal clear cell carcinoma

Chemokine	Fold change	P value
CCL1*	1.70	0.01
CCL2	1.23	0.165
CCL3*	3.20	< 0.001
CCL4*	5.93	< 0.001
CCL5*	8.30	< 0.001
CCL7	0.67	0.279
CCL8	0.86	0.448
CCL11	0.10	< 0.001
CCL13	1.65	0.278
CCL14	0.97	0.851
CCL15*	1.88	< 0.001
CCL16	0.99	0.965
CCL17*	2.49	0.002
CCL18*	90.77	0.01
CCL19	0.88	0.771
CCL20*	5.89	< 0.001
CCL21	0.70	0.392
CCL22*	2.13	< 0.001
CCL23	0.34	< 0.001
CCL24	1.31	0.389
CCL25*	6.91	0.011
CCL26	0.87	0.522
CCL27	0.90	0.586
CCL28*	3.06	< 0.001
CXCL1	1.48	0.355
CXCL2*	2.03	0.019
CXCL3	0.88	0.628
CXCL4	2.51	0.144
CXCL5*	20.41	0.006
CXCL6	0.79	0.523
CXCL7	1.98	0.284
CXCL8	0.79	0.542
CXCL9*	14.56	< 0.001
CXCL10*	11.93	< 0.001
CXCL11*	14.10	< 0.001
CXCL12	0.40	< 0.001
CXCL13*	17.90	0.001
CXCL14*	1.60	0.047
CXCL16*	2.05	< 0.001
CXCL17	0.41	0.344
CX3CL1*	2.15	< 0.001

*: The asterisk indicates chemokines with fold change >1 and P value < 0.05.

**Table 2:** The top 5 significant genes correlated with hub-chemokines in kidney renal clear cell carcinoma

Chemokine	Correlated genes
CCL1	ATXN2L, SYNGR3, KCNN4, RGL4, FBXW10B
CCL4	CCL3, CCL4L1, CRTAM, SIRPG, GZMA
CCL5	CD3D, CD3E, GZMA, CST7, CD27
CCL20	CXCL2, FHL2, ICAM1, RELB, CXCL1
CCL25	SYNGR3, PMCH, TNS4, DUSP2, PTPN7
CCL28	KISS1R, ANG, CDCA7L, HOXA4, ALDOC
CXCL2	CXCL3, NFKBIZ, CXCL1, ZC3H12A, CXCL8
CXCL5	CXCL6, TGFBI, CTHRC1, MMP7, IGDCC4
CXCL9	CXCL11, CXCL10, GBP5, TIGIT, CD3G
CXCL10	CXCL11, CXCL9, GBP1, GBP5, GBP1P1
CXCL11	CXCL10, CXCL9, GBP1, GBP5, GBP1P1
CXCL13	TIGIT, GZMK, SIRPG, CD27, SH2D1A
CX3CL1	AQP1, KANK3, BOC, NPR3, NAT8B

**Table 3:** COX regression analysis of immune cells for survival of kidney renal clear cell carcinoma

Immune cell	Hazard ratio	95%CIs	P value
B cell	0.549	0.02-13.57	0.71
CD8+ Tcell	0.175	0.04-0.83	0.03
CD4+ Tcell	0.592	0.04-8.9	0.71
Macrophage	0.062	0.01-0.64	0.02
Neutrophil	24.809	0.39-1582.75	0.13
Dendritic cell	3.062	0.52-18.13	0.22
