# Supplementary material for: A novel prognostic signature of chemokines for survival and immune infiltration in kidney renal clear cell carcinoma
Source: Int J Med Sci. 2023 Jun 19;20(8):1046–59. doi: 10.7150/ijms.84940 (PMC10357446; doi:10.7150/ijms.84940)
Supplement: Supplementary file 1 — Supplementary figures. [file ijmsv20p1046s1.pdf]

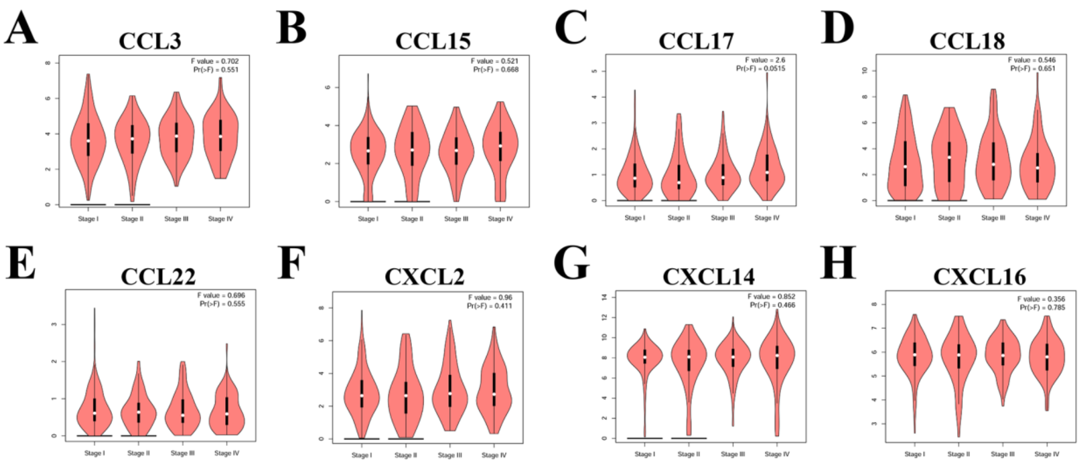

Supplementary Figure S1. The correlation of chemokines with different pathological stages of KIRC.

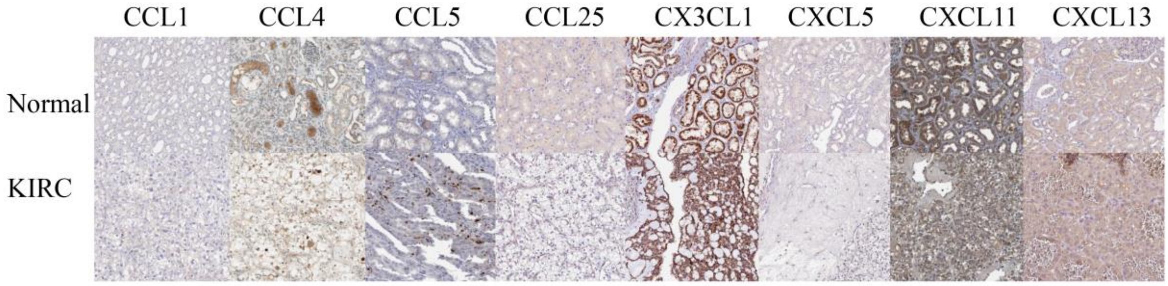

Supplementary Figure S2. The immunohistochemical (IHC) staining of chemokines in KIRC tumor tissues in the Human Protein Atlas (HPA) database.

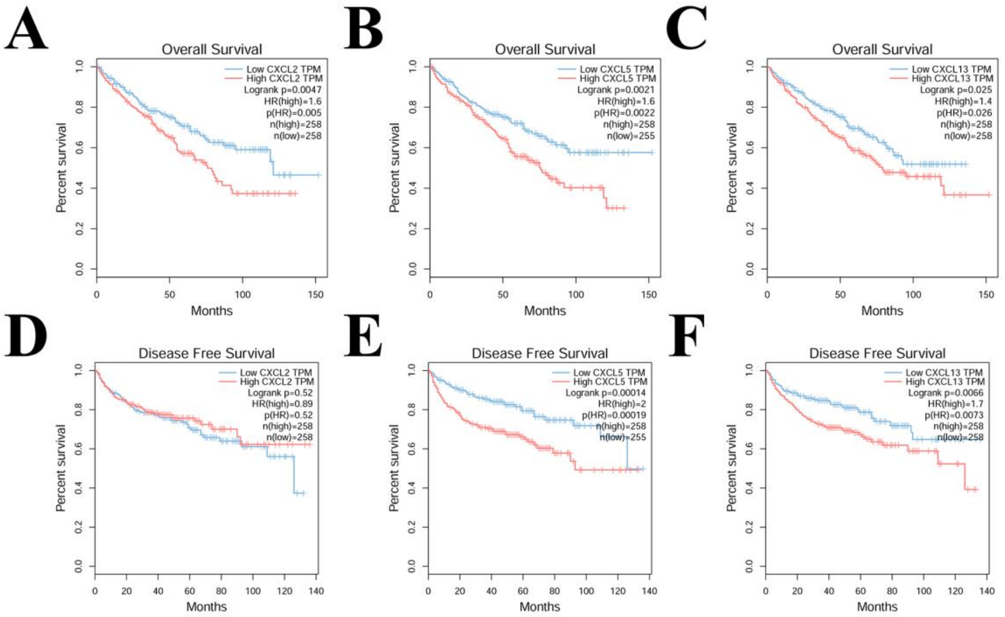

Supplementary Figure 3. **Prognosis analysis of chemokines in KIRC**. The overall survival curves of (A) CXCL2, (B) CXCL5 and (C) CXCL13 were plotted. The disease-free survival curves of (D) CXCL2, (E) CXCL5 and (F) CXCL13 were plotted.

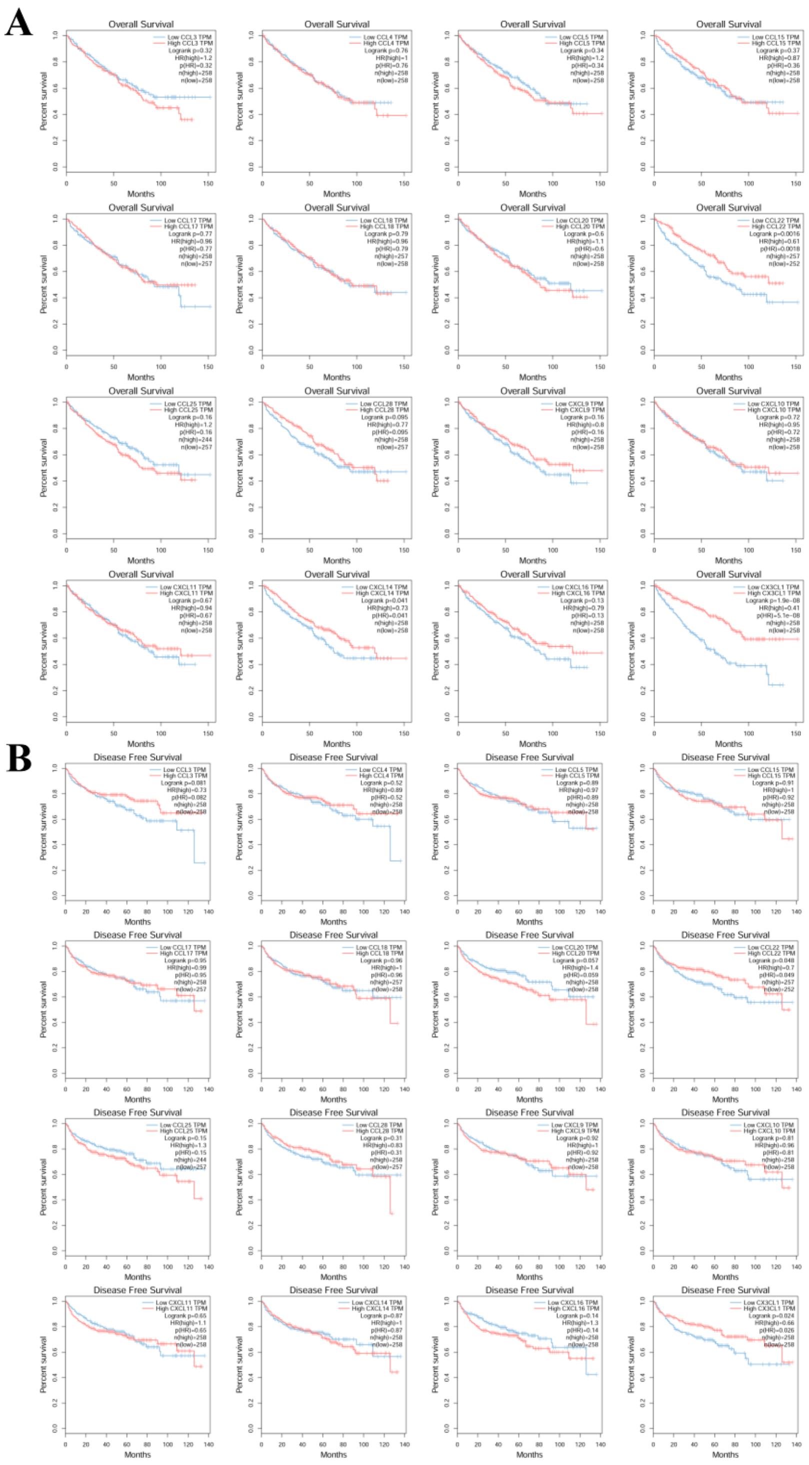

Supplementary Figure S4. Prognosis analysis of chemokines in KIRC. The curves of (A) overall survival and disease-free survival of chemokines in KIRC.

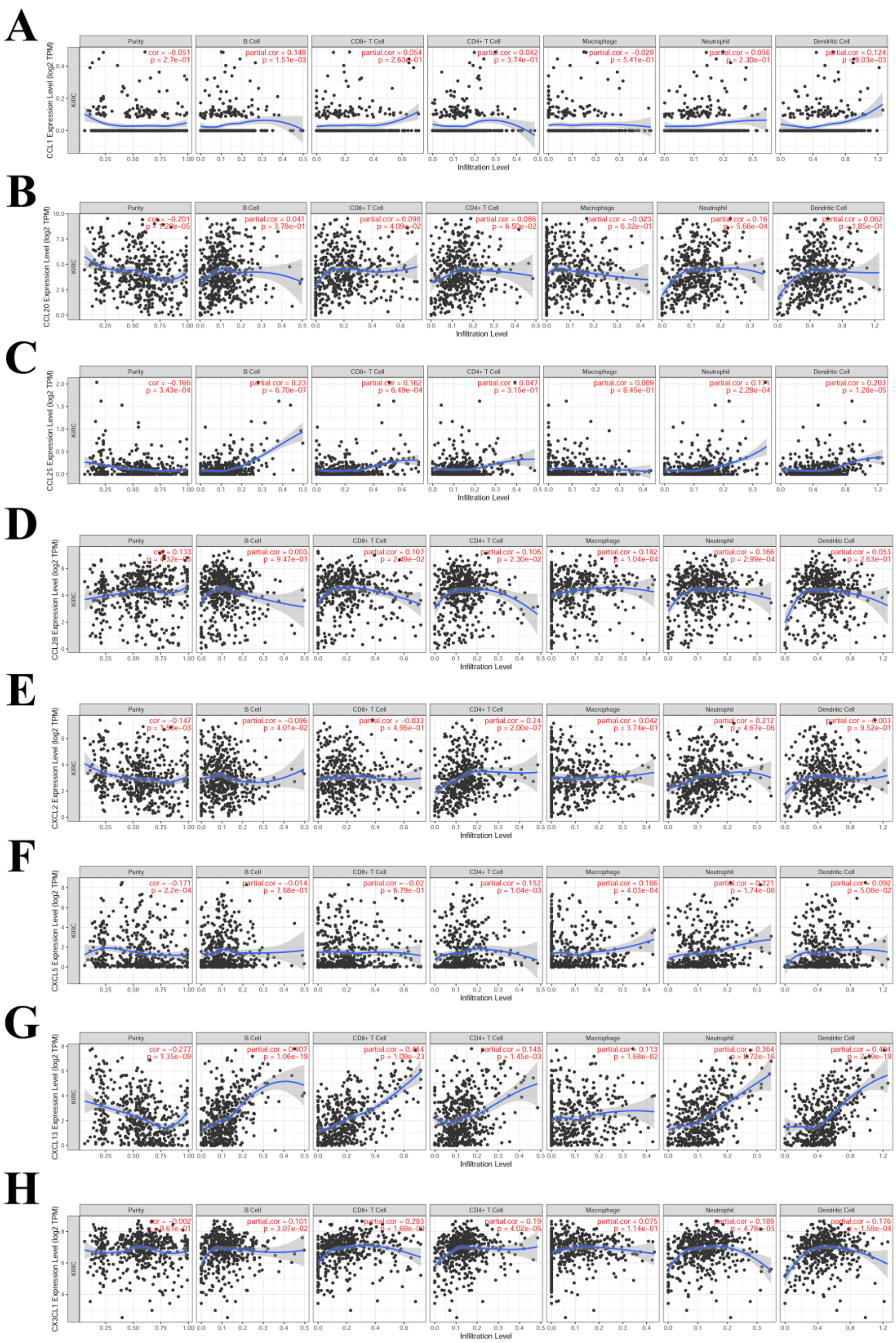

Supplementary Figure S5. Correlations analysis between hub-chemokines and different types of immune infiltration. Scatter plots of (A) CCL1, (B) CCL20, (C) CCL25, (D) CCL28, (E) CXCL2, (F) CXCL5, (G) CXCL13 and (H) CX3CL1.
